# Supplementary material for: Microbiota effects and predictors of Lactobacillus crispatus colonization after treatment with a vaginal live biotherapeutic: results from a randomized, double-blinded, placebo-controlled trial
Source: medRxiv. 2025 Aug 21:2025.08.18.25333897. Preprint. [Version 1] doi: 10.1101/2025.08.18.25333897 (PMC12393677; doi:10.1101/2025.08.18.25333897)
Supplement: Supplementary file 1 [file NIHPP2025.08.18.25333897V1-supplement-1.pdf]

medRxiv preprint doi: https://doi.org/10.1101/2025.08.18.25333897; this version posted August 21, 2025. The copyright holder for this preprint (which was not certified by peer review) is the author/funder, who has granted medRxiv a license to display the preprint in perpetuity.
It is made available under a CC-BY 4.0 International license .

Supplementary Tables and Figures

Table S1

	At least one visit	Pre-MTZ	Post-MTZ	Week 4	Week 8	Week 12	Week 24	All planned in-person visits
**Total**	211	208	203	181	172	179	165	139
**LACTIN-V**	142	140	138	124	120	123	113	99
**Placebo**	69	68	65	57	52	56	52	40

Number of participants in each study arm (rows) with available 16S rRNA gene sequencing data at any, each, or all planned in-person visits (unscheduled clinic visits not shown).

medRxiv preprint doi: https://doi.org/10.1101/2025.08.18.25333897; this version posted August 21, 2025. The copyright holder for this preprint (which was not certified by peer review) is the author/funder, who has granted medRxiv a license to display the preprint in perpetuity.
It is made available under a CC-BY 4.0 International license .

Table S2

	% (n) [both arm]	% (n) [LACTIN-V]	% (n) [Placebo]
≥ 50% *L. iners*	52 % (106)	52 % (72)	52 % (34)
≥ 50% *L. jensenii*	8 % (16)	9 % (13)	5 % (3)
≥ 50% *L. crispatus*	1 % (2)	1 % (1)	2 % (1)
≥ 50% *Lactobacillus* (no individual species ≥ 50%)	13 % (27)	14 % (20)	11 % (7)
< 50% *Lactobacillus*	26 % (52)	23 % (32)	31 % (20)

At the post-MTZ visit, number and proportion of samples dominated by one of the top *Lactobacillus* species (top 3 rows), a mixture of *Lactobacillus* (4th row), or by non-*Lactobacillus* species in both arms (2nd column), the LACTIN-V arm (3rd column), or the placebo arm (4th column).

medRxiv preprint doi: https://doi.org/10.1101/2025.08.18.25333897; this version posted August 21, 2025. The copyright holder for this preprint (which was not certified by peer review) is the author/funder, who has granted medRxiv a license to display the preprint in perpetuity.
It is made available under a CC-BY 4.0 International license .

Table S3

	Clinical diagnosis	
**Microbiota outcome** (16S rRNA sequencing-based)	no rBV n (%)	rBV n (%)
< 50% *Lactobacillus*	162 (50 %)	164 (50 %)
≥ 50% *Lactobacillus, <50% L. crispatus*	192 (99 %)	2 (1 %)
≥ 50% *L. crispatus*	209 (100 %)	0 (0 %)

Number of visits (n) at which participants did not (left column) or did (right column) meet diagnostic criteria for recurrent BV (rBV). Participants are shown according to microbiota category. Percentages sum to 100% for each category.

medRxiv preprint doi: https://doi.org/10.1101/2025.08.18.25333897; this version posted August 21, 2025. The copyright holder for this preprint (which was not certified by peer review) is the author/funder, who has granted medRxiv a license to display the preprint in perpetuity.
It is made available under a CC-BY 4.0 International license .

Table S4

	Category at **week 12**			
Category at week 4	≥ 50% *L. crispatus*	≥ 50% *Lactobacillus*, < 50% *L. crispatus*	< 50% *Lactobacillus*	Total
≥ 50% *L. crispatus*	25	14	12	51
≥ 50% *Lactobacillus*, < 50% *L. crispatus*	6	12	8	26
< 50% *Lactobacillus*	4	4	30	38
Total	35	30	50	115

Contingency table showing the correspondence between Week 4 and Week 12 microbiota categories for LACTIN-V recipients with available data from both visits.

medRxiv preprint doi: https://doi.org/10.1101/2025.08.18.25333897; this version posted August 21, 2025. The copyright holder for this preprint (which was not certified by peer review) is the author/funder, who has granted medRxiv a license to display the preprint in perpetuity.
It is made available under a CC-BY 4.0 International license .

Table S5

	Category at **week 24**			
Category at week 4	≥ 50% *L. crispatus*	≥ 50% *Lactobacillus*, < 50% *L. crispatus*	< 50% *Lactobacillus*	Total
≥ 50% *L. crispatus*	27	9	11	47
≥ 50% *Lactobacillus*, < 50% *L. crispatus*	6	6	11	23
< 50% *Lactobacillus*	5	10	22	37
Total	38	25	44	107

Contingency table showing the correspondence between Week 4 and Week 24 microbiota categories for LACTIN-V recipients with available data from both visits.

medRxiv preprint doi: https://doi.org/10.1101/2025.08.18.25333897; this version posted August 21, 2025. The copyright holder for this preprint (which was not certified by peer review) is the author/funder, who has granted medRxiv a license to display the preprint in perpetuity.
It is made available under a CC-BY 4.0 International license .

Table S6

Block	Variable	Description
Demographics	Age	Age of the participant at enrollment.
	N past BV	Number of past BV episodes (coded as a number on the following scale: (1) None, (2) 1-2, (3) Unknown, (4) 3-4, (5) 5 or more).
	Education level	Education level of the participant (coded as a number on the following scale: (1) Did not complete high school, (2) Completed high school, (3) Completed junior college, (4) Completed college (undergraduate degree), (5) Completed graduate degree).
	Race	Self-declared race of the participant (Categories with few participants were merged into "Other").
Vag. env. pre-MTZ	α diversity	Shannon diversity index of baseline (pre-MTZ) microbiota, computed on taxa proportions quantified from 16S rRNA gene sequencing data.
	pH	Vaginal pH at the pre-MTZ visit, measured at study sites.
Microbiota pre-MTZ		Pre-MTZ microbiota composition described as topic proportions.
Cytokine (r) pre-MTZ		Pre-MTZ cytokine residual levels. These values are computed as the difference between the transformed cytokine/chemokines levels and their predicted value based on microbiota composition expressed as topic proportions.
Coloniz. cat. prev. v.		Colonization category at the previous visit. One of these mutually exclusive categories: ≥ 50% *L. crispatus*, ≥ 50% *Lactobacillus* and < 50% *L. crispatus*, or < 50% *Lactobacillus*.
Vag. env. prev. v.	α diversity (r)	Difference between the Shannon diversity index at the previous visit (w.r.t the visit of the response variable), computed on taxa proportions quantified from 16S rRNA gene sequencing data, and its expected value based on colonization category at the same visit.
	pH (r)	Difference between vaginal pH at the previous visit, measured at study sites, and its expected value based on colonization category at the same visit.
	log10(bact. load)	Log_{10} total bacterial load at the previous visit, quantified by qPCR.
Microbiota (r) prev. v.		Difference between the observed microbiota composition at the previous visit described as topic proportions and the average microbiota composition within each colonization category.
Cytokines (r) prev. v.		Cytokine residual levels at the previous visit. These values are computed as the difference between the transformed cytokine/chemokines levels and their predicted value based on microbiota composition expressed as topic proportions.
Birth control	Non-hormonal	A binary variable indicating whether participants reported exclusively using one of the following non-hormonal birth control methods including abstinence, use of condoms, or fertility awareness methods.
	IUD (Hormonal)	Same as above for hormonal intra-uterine device.
	IUD (Non-hormonal)	Same as above for non-hormonal intra-uterine device (copper IUD).

medRxiv preprint doi: https://doi.org/10.1101/2025.08.18.25333897; this version posted August 21, 2025. The copyright holder for this preprint (which was not certified by peer review) is the author/funder, who has granted medRxiv a license to display the preprint in perpetuity.
It is made available under a CC-BY 4.0 International license .

	Combined	A binary variable indicating whether participants reported using a combination of hormonal contraceptives or switched contraceptive method between the last visit and this one.
	P only	A binary variable indicating whether participants reported exclusively using progestin-only hormonal contraceptive, including the pill, patches, and implants.
	unknown	A binary variable indicating whether a participant's birth control is unknown (missing data). Each participant belongs to only one of each birth control category.
Sexual behavior	N new partners	Number of new sexual partners since the last visit.
	Any condomless sex	A binary flag indicating whether the participant reported any sexual intercourse without condoms since the last visit.
	Any condom sex	A binary flag indicating whether the participant reported any sexual intercourse with condoms since the last visit.
Perturbations	N douching	Number of days participant reported any douching episode since the last visit.
	N bleeding	Number of days participant reported vaginal bleeding since the last visit.
Antibiotics	N vag. abx	Number of days participant reported using vaginal antibiotics since the previous visit.
	N oral abx	Number of days participant reported taking oral antibiotics since the previous visit.
Adherence	N missed doses	Difference between the number of LACTIN-V doses that should have been taken between the previous visit and the current visit if the study protocol had been strictly followed and the actual number of doses that were taken.
	Days since last dose	Number of days since the most recent LACTIN-V dose was taken.

medRxiv preprint doi: https://doi.org/10.1101/2025.08.18.25333897; this version posted August 21, 2025. The copyright holder for this preprint (which was not certified by peer review) is the author/funder, who has granted medRxiv a license to display the preprint in perpetuity.
It is made available under a CC-BY 4.0 International license .

Figure S1

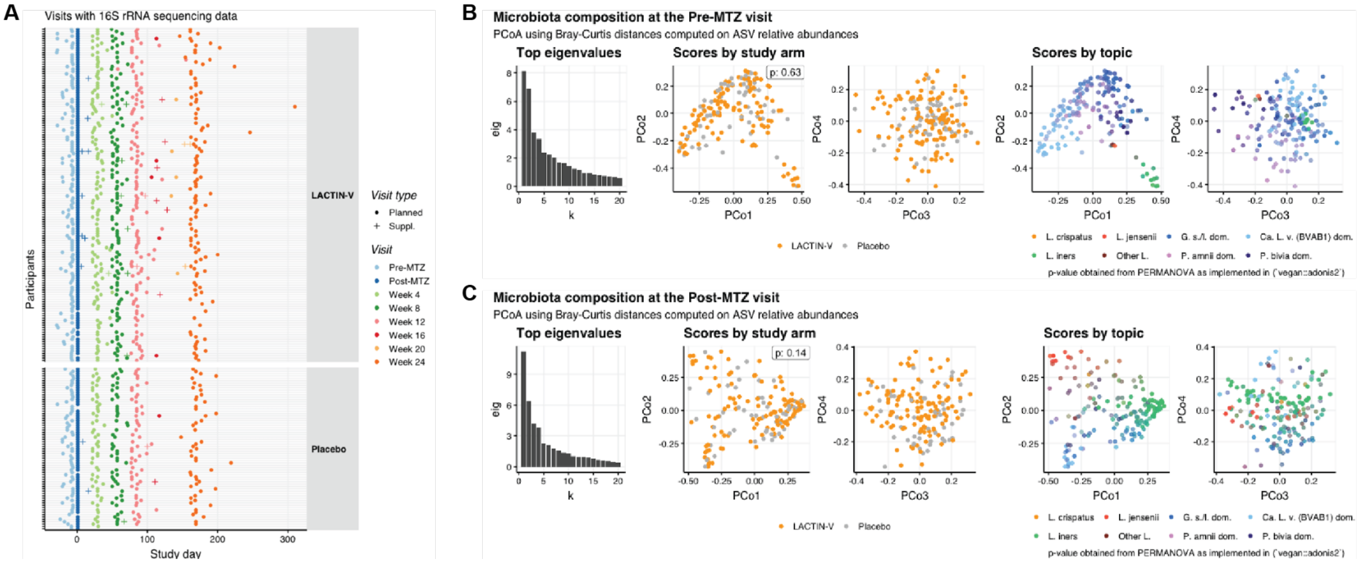

A. Time-line of collected swabs from which 16S rRNA gene sequencing data could be generated.
B. Balance between treatment arms in terms of microbiota composition at the pre-MTZ visit. The left panel shows the top 20 eigenvalues of the PCoA (Principal Coordinate Analysis) performed on the Bray-Curtis dissimilarity matrix, computed from the relative abundances of ASVs. The next two panels show the PCoA scores (projection of the samples, each dot is a sample) colored by the intervention arm for the first 4 principal coordinates. The *p*-value in the upper right corner corresponds to the PERMANOVA test on the study arm. The right panels show the same projections as the middle panels, but samples are here colored by topic relative abundance (**Figure 2A-B**).
C. Balance between treatment arms in terms of microbiota composition at the post-MTZ visit, labeled and arranged as for **Figure S1B**.

medRxiv preprint doi: https://doi.org/10.1101/2025.08.18.25333897; this version posted August 21, 2025. The copyright holder for this preprint (which was not certified by peer review) is the author/funder, who has granted medRxiv a license to display the preprint in perpetuity.
It is made available under a CC-BY 4.0 International license .

Figure S2

medRxiv preprint doi: https://doi.org/10.1101/2025.08.18.25333897; this version posted August 21, 2025. The copyright holder for this preprint (which was not certified by peer review) is the author/funder, who has granted medRxiv a license to display the preprint in perpetuity.
It is made available under a CC-BY 4.0 International license .

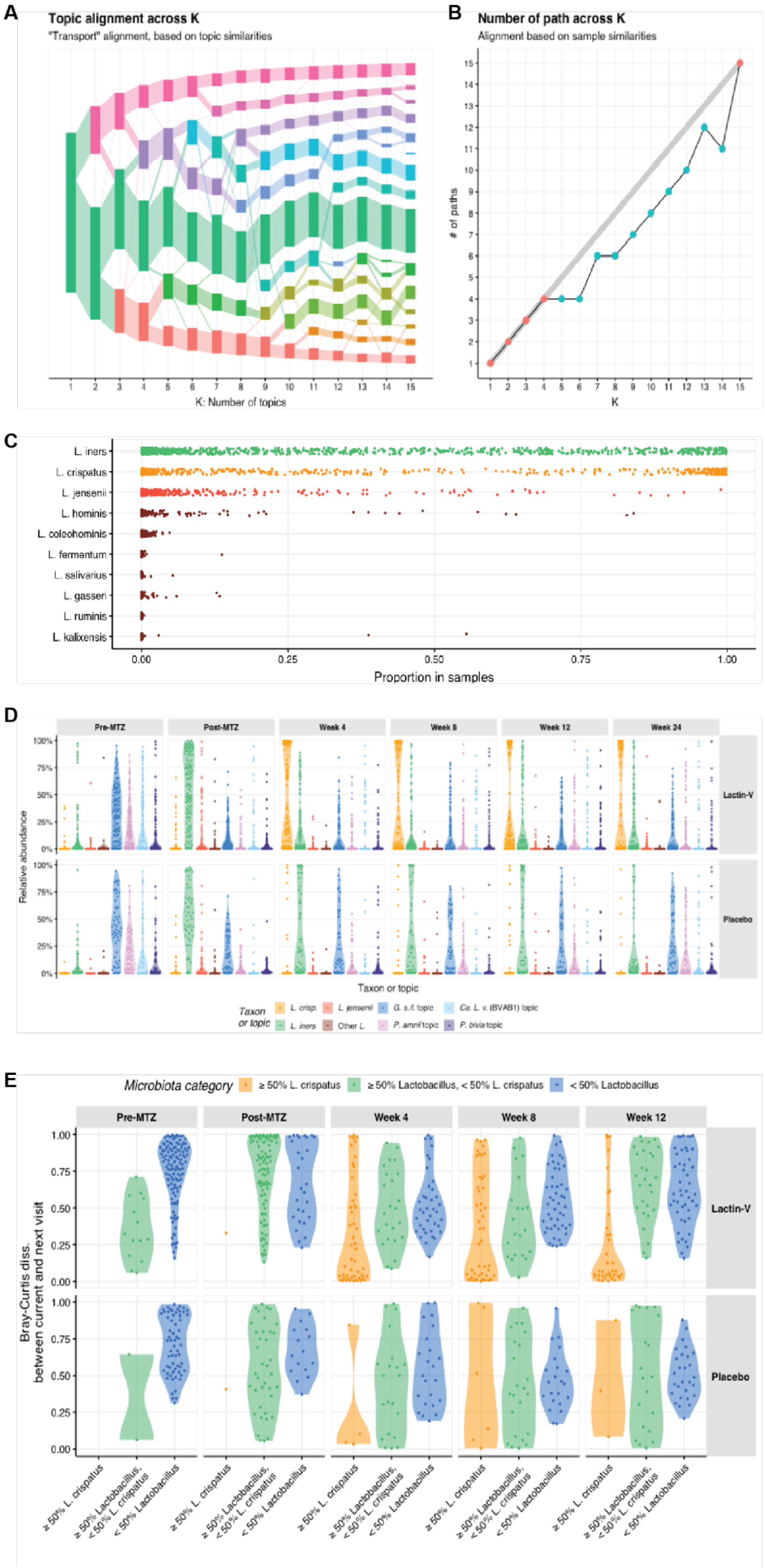

1335

medRxiv preprint doi: https://doi.org/10.1101/2025.08.18.25333897; this version posted August 21, 2025. The copyright holder for this preprint (which was not certified by peer review) is the author/funder, who has granted medRxiv a license to display the preprint in perpetuity.
It is made available under a CC-BY 4.0 International license .

A. "Transport" alignment between topics identified by LDA models fitted on the non-*Lactobacillus* taxa counts for varying total number of topics (K, x-axis). Each rectangle represents a topic; the height of that rectangle represents the summed prevalence of that topic in the population, and the size of the connection between topics is proportional to the degree of similarity between the composition of two given topics.

B. The number of paths (y-axis) identified at each resolution (K, the number of topics considered by the LDA models, x-axis) from the "product" alignment of topics; for details see[77]. A path identifies a series of topics with high alignment scores throughout resolutions. A plateau in the number of paths (here at K = 4) suggests that the 4-topic model is the model that identifies most robust topics (i.e., topics that remain similar across resolution despite the introduction of new, potentially spurious, topics).

C. Proportion (x-axis) of each *Lactobacillus* species (y-axis) detected across all samples. Species with a relative abundance > 50% in at least 10 samples made up their own topic.

D. Relative abundance of taxon or topic (x-axis, colors) in each arm at each visit.

E. Microbiota stability by arm, visit, and microbiota category. Stability is quantified using the Bray-Curtis dissimilarity, described as relative abundances at the ASV level at the participant's current visit (vertical panels) and subsequent scheduled visit. At each visit, participants are grouped into one of the three categories (colors, x-axis) defined earlier (*L. crispatus*-dominant, *Lactobacillus*-dominant but low-level *L. crispatus*, and low-level *Lactobacillus*).

medRxiv preprint doi: https://doi.org/10.1101/2025.08.18.25333897; this version posted August 21, 2025. The copyright holder for this preprint (which was not certified by peer review) is the author/funder, who has granted medRxiv a license to display the preprint in perpetuity.
It is made available under a CC-BY 4.0 International license .

1355 **Figure S3**

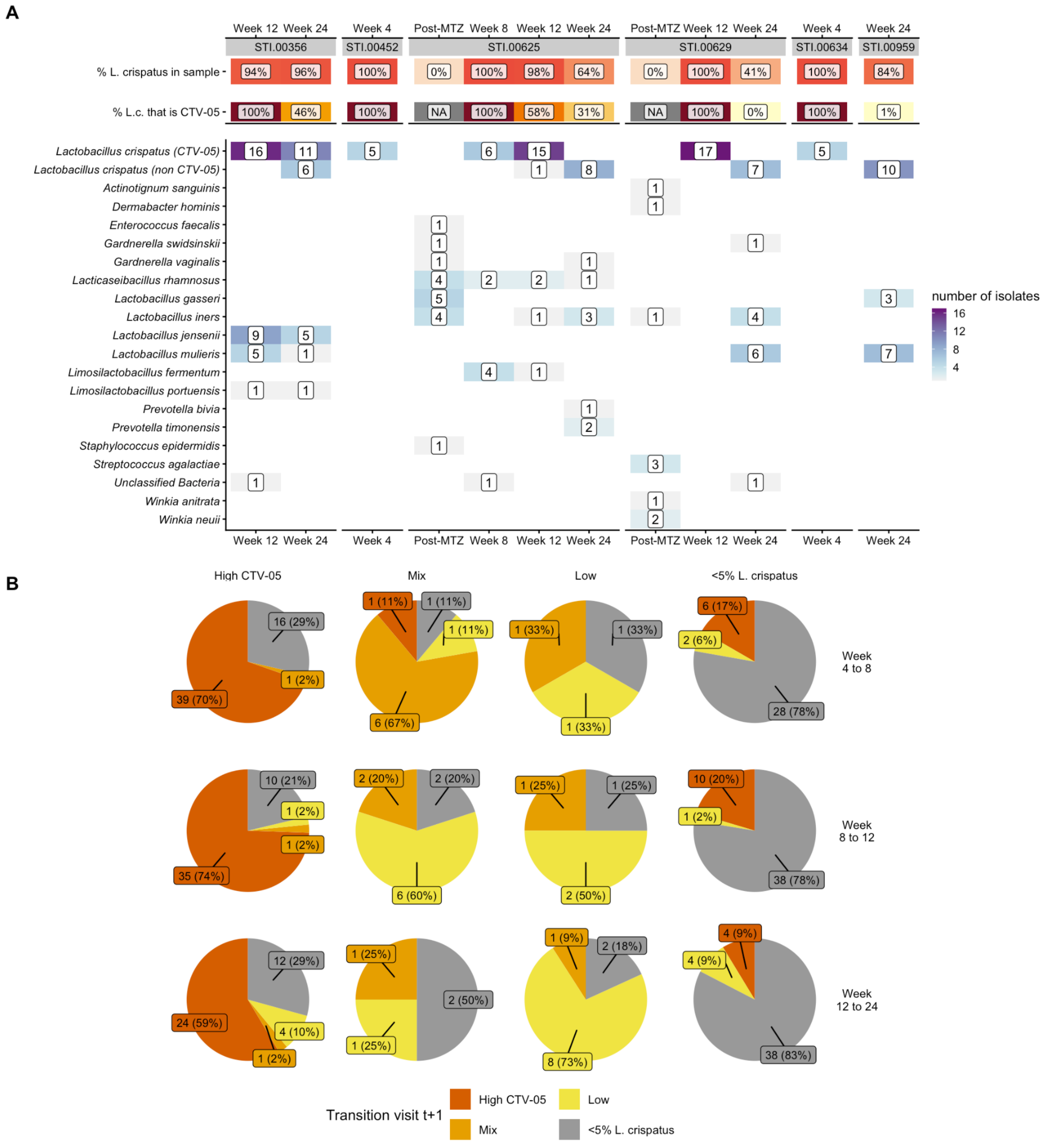

1356

medRxiv preprint doi: https://doi.org/10.1101/2025.08.18.25333897; this version posted August 21, 2025. The copyright holder for this preprint (which was not certified by peer review) is the author/funder, who has granted medRxiv a license to display the preprint in perpetuity.
It is made available under a CC-BY 4.0 International license .

A. Results of targeted isolations from LACTIN-V trial samples, showing source participant, visit number, sample *L. crispatus* relative abundance as estimated by 16S rRNA gene sequencing, CTV-05 proportional strain abundance, and the numbers and taxonomic assignments of genome-sequenced isolates from each sample. *L. crispatus* isolates are separated by whether genome sequencing showed them to be CTV-05 or non-CTV-05 (native) strains. Multiple isolates of genotypically identical strains (including of *L. crispatus* isolates) were obtained from some samples.

B. Pie charts for each Visit t strain category from **Figure 3F** summarizing *L. crispatus* strain category frequencies at Visit t+1, shown individually for each Visit t.

medRxiv preprint doi: https://doi.org/10.1101/2025.08.18.25333897; this version posted August 21, 2025. The copyright holder for this preprint (which was not certified by peer review) is the author/funder, who has granted medRxiv a license to display the preprint in perpetuity.
It is made available under a CC-BY 4.0 International license .

Figure S4

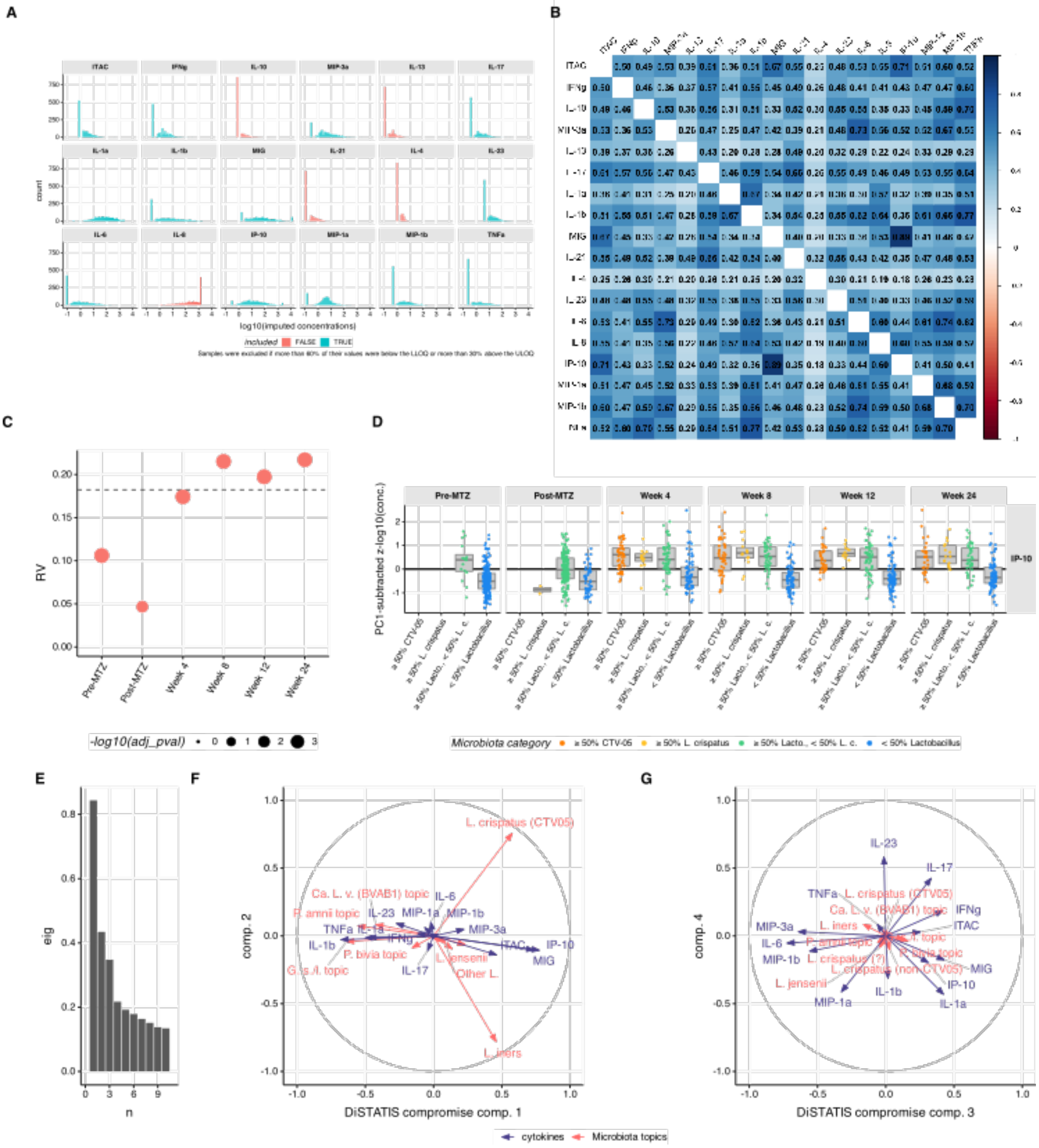

medRxiv preprint doi: https://doi.org/10.1101/2025.08.18.25333897; this version posted August 21, 2025. The copyright holder for this preprint (which was not certified by peer review) is the author/funder, who has granted medRxiv a license to display the preprint in perpetuity.
It is made available under a CC-BY 4.0 International license .

A. Distribution of unadjusted cytokine and chemokine concentrations within the dataset, with values below the lower limit of quantification (LLOQ) imputed at half the LLOQ and values above the upper limit of quantification (ULOQ) imputed at the ULOQ. Analytes were excluded from further analysis if >60% of values were below the LLOQ or >30% were above the ULOQ.

B. Pair-wise Pearson correlation coefficients between log10-transformed concentrations of each cytokine. All correlations were significant ($p < 0.05$).

C. RV coefficients (y-axis) between microbiota composition described in terms of topic proportions and cytokine/chemokine transformed concentrations at each visit (x-axis). Permutation test *p*-values were adjusted for multiple testing to control for the false discovery rate. The size of the dot is inversely proportional to the $\log_{10}$ of the associated adjusted p-value. The horizontal dashed line provides the value of the RV coefficient when computed on all visits combined.

D. IP-10 adjusted $\log_{10}$(concentrations) (y-axis) by microbiota composition categories (x-axis) at each visit (horizontal panels). Microbiota categories are mutually exclusive such that the first category (≥ 50% CTV-05) encompasses samples in which the relative abundance of CTV-05 (within the overall microbiota) is larger or equal to 50%; the second category encompasses samples with ≥50% total *L. crispatus* but <50% CTV-05 relative abundance within the microbiota; the third category includes samples in which total *L. crispatus* relative abundance is <50%, but the total *Lactobacillus* relative abundance is ≥50%, and the remaining category includes samples with <50% *Lactobacillus*. Participants from both arms are included.

E. DISTATIS screeplot from repeated DiSTATIS analysis, similar to analysis in **Figure 4D** but differentiating between CTV-05 and summed other *L. crispatus* strains. Eigenvalues (a.u.) of DISTATIS compromise for the first 10 latent components are shown.

medRxiv preprint doi: https://doi.org/10.1101/2025.08.18.25333897; this version posted August 21, 2025. The copyright holder for this preprint (which was not certified by peer review) is the author/funder, who has granted medRxiv a license to display the preprint in perpetuity.
It is made available under a CC-BY 4.0 International license .

F. DISTATIS correlation circle for the analysis in **Figure S4E**, showing the 1st and 2nd latent components (similar to **Figure 4E**).
G. DISTATIS correlation circle for the analysis in **Figure S4E**, showing the 3rd and 4th latent components (similar to **Figure 4F**).

medRxiv preprint doi: https://doi.org/10.1101/2025.08.18.25333897; this version posted August 21, 2025. The copyright holder for this preprint (which was not certified by peer review) is the author/funder, who has granted medRxiv a license to display the preprint in perpetuity.
It is made available under a CC-BY 4.0 International license .

Figure S5

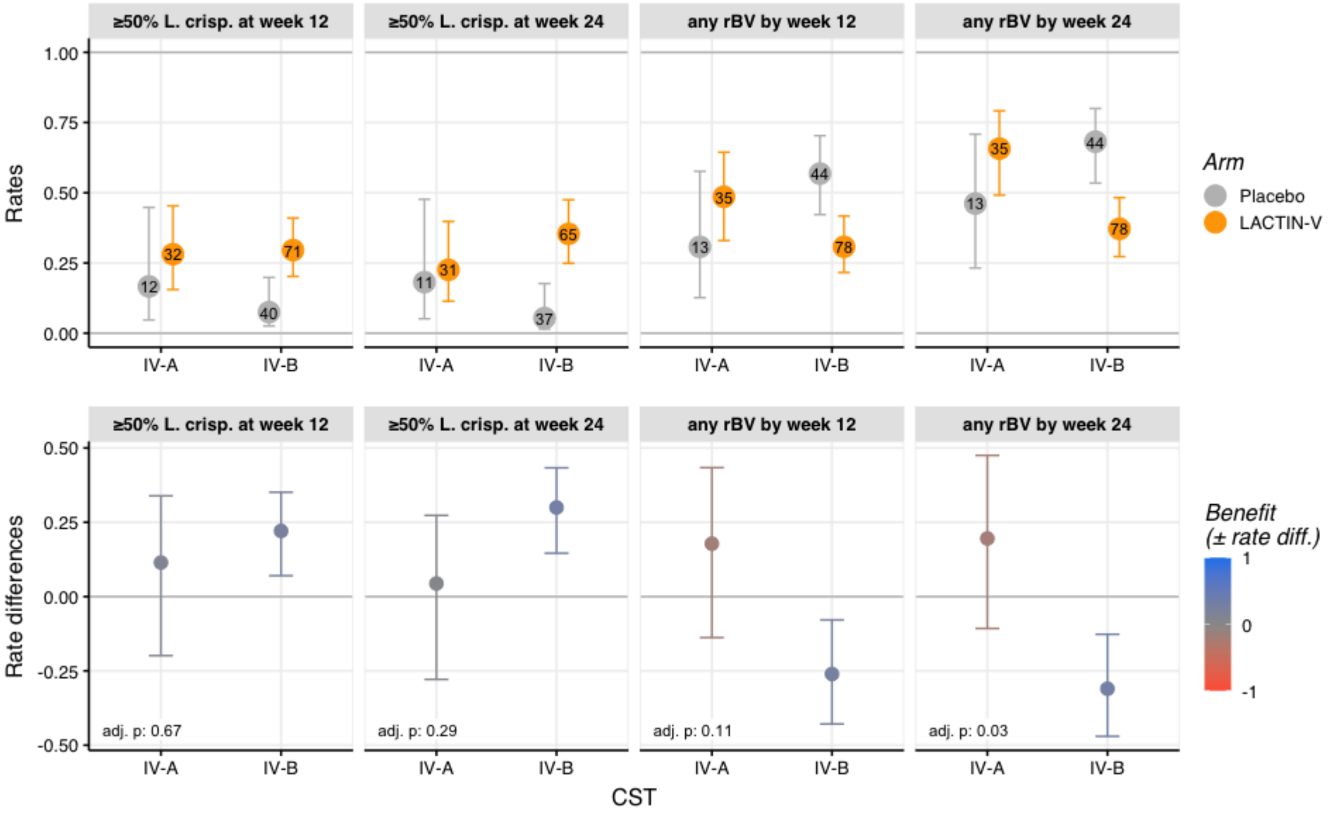

Rates and associated 95% CI of high-level *L. crispatus* colonization (≥50% of relative abundance) (y-axis) at week 12 or 24 (left two panels, top row) or rBV by Week 12 or 24 (right two panels, top row) by pre-MTZ CST in each arm (colors), and the corresponding rate differences and 95% CI between arms (bottom row), analyzed as in **Figure 5C-F**. Analysis is restricted to CSTs with at least two participants in each arm (*i.e.,* CSTs IV-A and IV-B). The color scale in the bottom row shows the degree of benefit (defined as the difference in rates between the two arms) in achieving *L. crispatus*-dominance (left panels) or reducing rBV (right panels), with blue indicating benefit with LBP treatment and red indicating benefit with placebo. *P*-values adjusted for multiple testing using the Benjamini-Hochberg correction of the analysis of deviance test for heterogeneity in treatment effects when stratifying participants by CST

medRxiv preprint doi: https://doi.org/10.1101/2025.08.18.25333897; this version posted August 21, 2025. The copyright holder for this preprint (which was not certified by peer review) is the author/funder, who has granted medRxiv a license to display the preprint in perpetuity.
It is made available under a CC-BY 4.0 International license .

at the pre-MTZ visit. The centroid definition of CST IV-A includes *Ca*. Lachnocurva vaginae (BVAB1) as a predominant taxon while CST IV-B has higher abundance of *Gardnerella* and both share moderate *Prevotella* abundance[57].

medRxiv preprint doi: https://doi.org/10.1101/2025.08.18.25333897; this version posted August 21, 2025. The copyright holder for this preprint (which was not certified by peer review) is the author/funder, who has granted medRxiv a license to display the preprint in perpetuity.
It is made available under a CC-BY 4.0 International license .

Figure S6

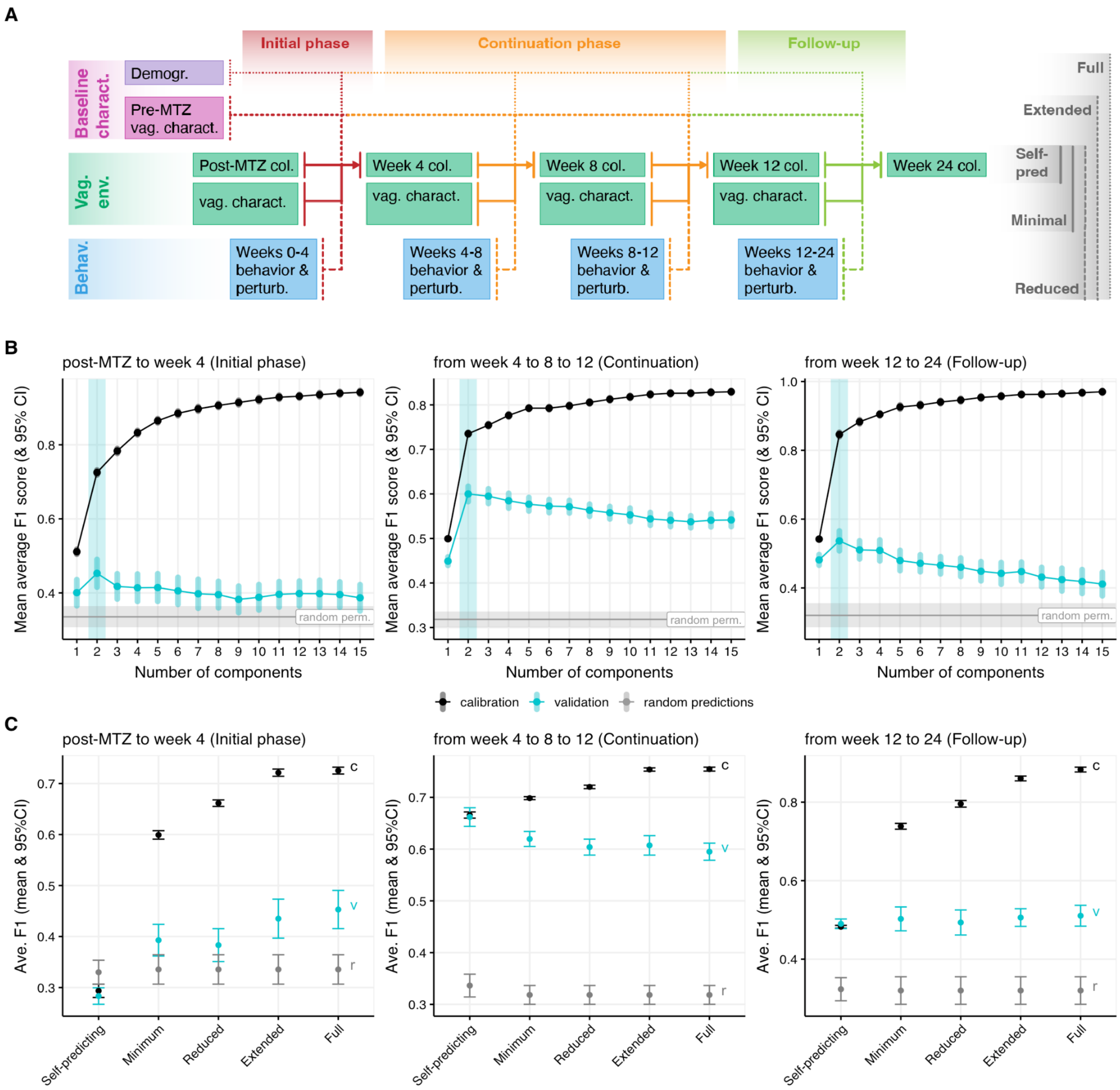

A. Diagram depicting MB-PLS-DA nested models. To assess whether blocks contributed to explaining colonization, we compared a series of nested models (far-right labels). The full model includes all blocks and variables, while the “self-predicting” model solely includes the microbiota categories at the previous visit.

medRxiv preprint doi: https://doi.org/10.1101/2025.08.18.25333897; this version posted August 21, 2025. The copyright holder for this preprint (which was not certified by peer review) is the author/funder, who has granted medRxiv a license to display the preprint in perpetuity.
It is made available under a CC-BY 4.0 International license .

B. Number of latent component selection by cross-validation for the initial phase (left), continuation phase (middle), and follow-up phase models (S6A). Mean (dots) and 95% CI (vertical segment) of the average F1 score (y-axis) obtained when including k latent components (x-axis) on 20 random calibration (black) or validation (turquoise) set. For reference, values obtained for random permutations of the response labels are shown by the horizontal gray line and band. The number of selected latent components is shown by the light turquoise vertical band (here k = 2 for all phases).

C. Contribution of nested models for the initial, continuation, and follow-up phase models. Mean and 95%CI of the average F1 score (y-axis) on the calibration (black, “c”), validation (turquoise, “v”), or for random predictions (gray, “r”) for each nested model (x-axis) including the number of latent components shown on the corresponding upper-panel.

medRxiv preprint doi: https://doi.org/10.1101/2025.08.18.25333897; this version posted August 21, 2025. The copyright holder for this preprint (which was not certified by peer review) is the author/funder, who has granted medRxiv a license to display the preprint in perpetuity.
It is made available under a CC-BY 4.0 International license .

Figure S7: Variables associated with microbiota categories in LACTIN-V recipients

medRxiv preprint doi: https://doi.org/10.1101/2025.08.18.25333897; this version posted August 21, 2025. The copyright holder for this preprint (which was not certified by peer review) is the author/funder, who has granted medRxiv a license to display the preprint in perpetuity.
It is made available under a CC-BY 4.0 International license .

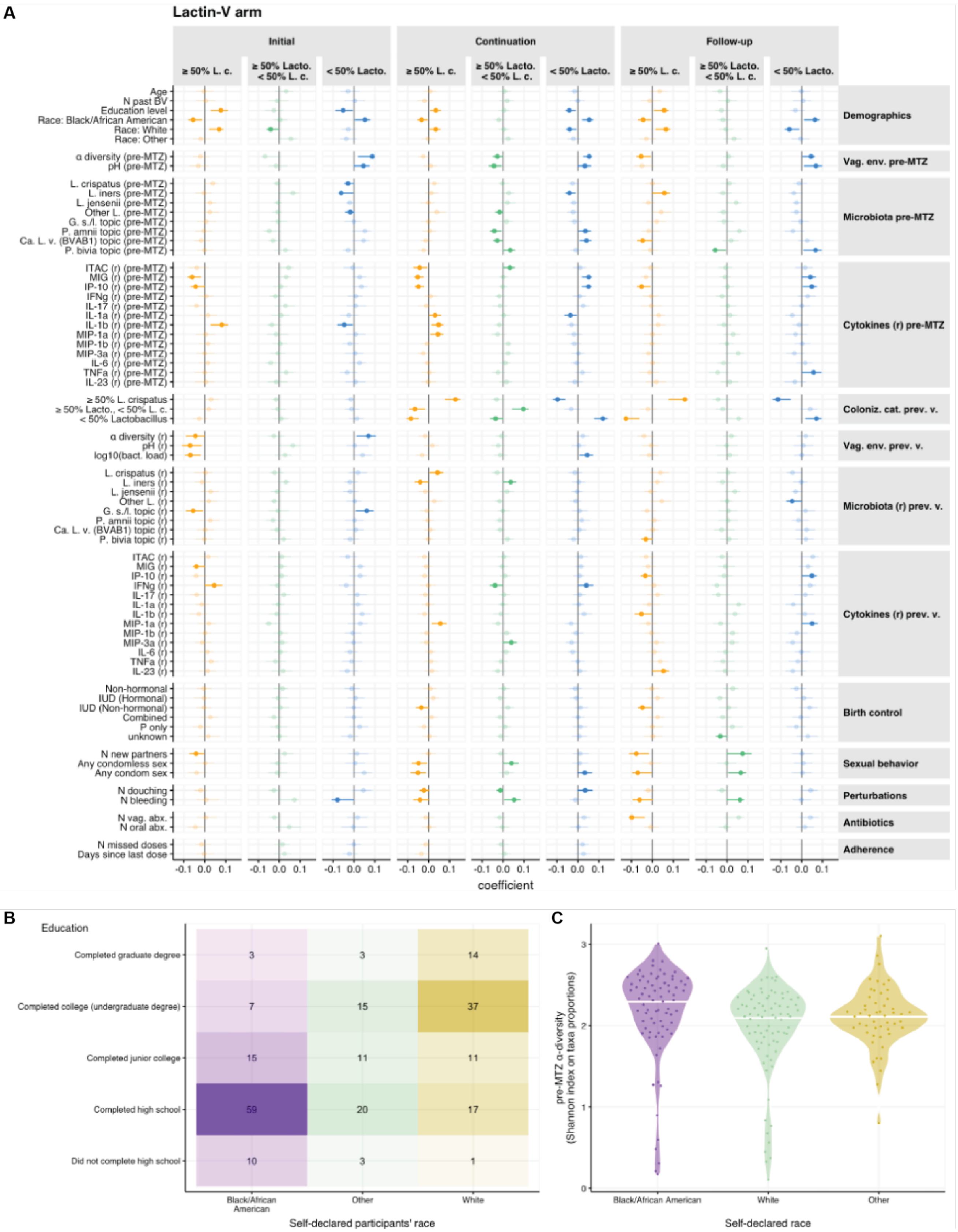

medRxiv preprint doi: https://doi.org/10.1101/2025.08.18.25333897; this version posted August 21, 2025. The copyright holder for this preprint (which was not certified by peer review) is the author/funder, who has granted medRxiv a license to display the preprint in perpetuity.
It is made available under a CC-BY 4.0 International license .

A. Coefficient of association (dots, x-axis) and associated bootstrapped 95% CI (horizontal segments) between explanatory variables (y-axis) and microbiota categories (colors, horizontal panels, see **Figure 6A**) for each phase of the trial (horizontal panels) for MB-PLS-DA models fitted on LBP recipient data (**Figure S6A**). Variables whose 95% CI include 0 are shown in transparent/lighter shades. Variables are shown grouped according to their assigned thematic blocks (**Figure 6A**, **Table S6**).

B. Self-reported education levels are significantly associated with self-reported race (χ^2 *p*-value < 0.01)

C. Pre-MTZ α-diversity is higher in Black or African American participants than in White participants (*p*-value of *t*-test on association coefficient < 0.05).

medRxiv preprint doi: https://doi.org/10.1101/2025.08.18.25333897; this version posted August 21, 2025. The copyright holder for this preprint (which was not certified by peer review) is the author/funder, who has granted medRxiv a license to display the preprint in perpetuity.
It is made available under a CC-BY 4.0 International license .

Figure S8: Multiblock analysis of factors associated with microbiota categories in placebo recipients

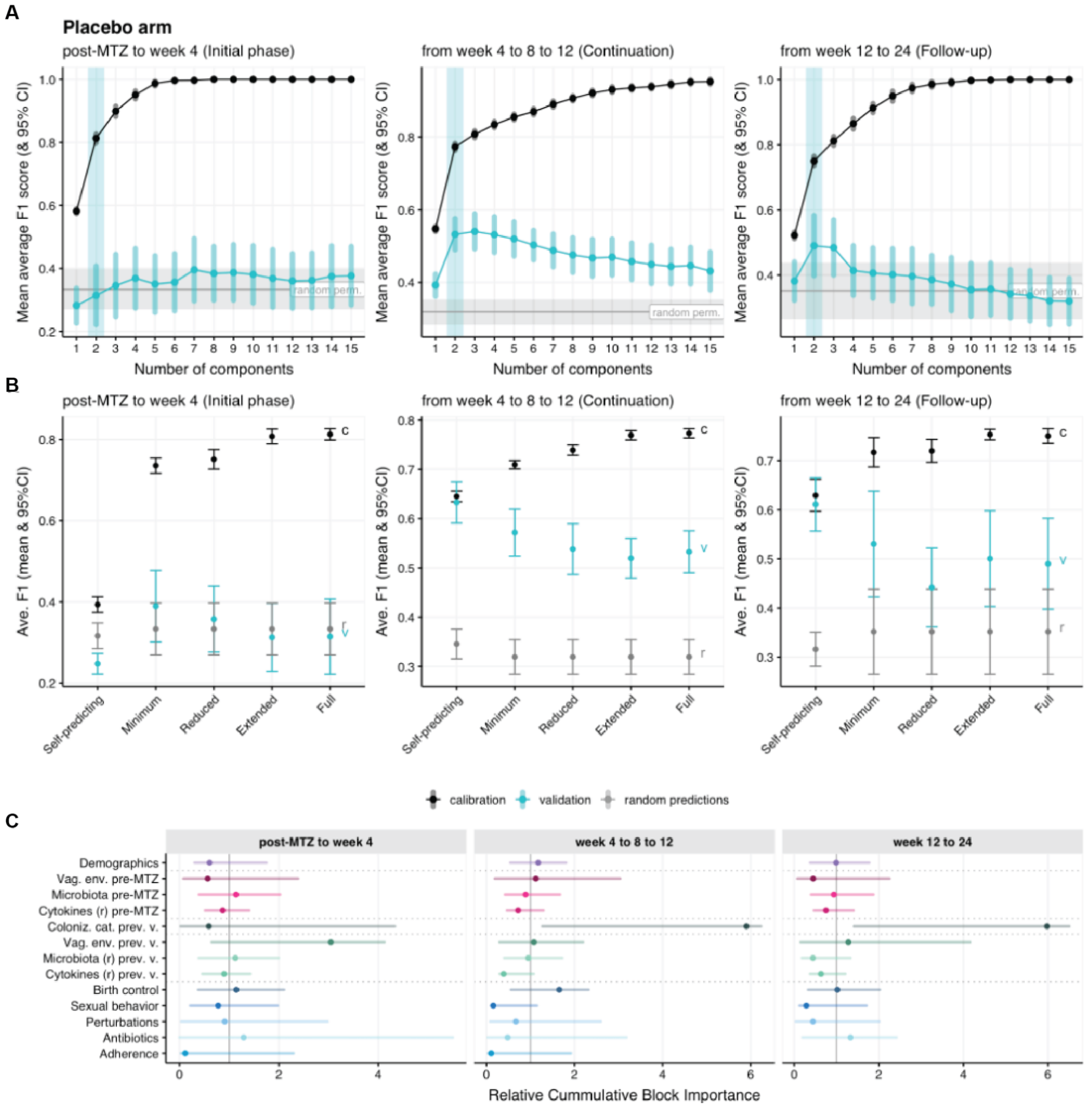

A. Number of latent component selection by cross-validation for the initial (left), continuation (middle), and follow-up phase (right) model fitted on the placebo arm data. Reads as **Figure S6B**.

medRxiv preprint doi: https://doi.org/10.1101/2025.08.18.25333897; this version posted August 21, 2025. The copyright holder for this preprint (which was not certified by peer review) is the author/funder, who has granted medRxiv a license to display the preprint in perpetuity.
It is made available under a CC-BY 4.0 International license .

B. Contribution of nested models fitted on the placebo arm data for the initial (left), continuation (missing) and follow-up phase (right). Reads as **Figure S6C.**

C. Relative cumulative importance indices in the placebo arm (x-axis, Methods) for each block (y-axis, color) for the initial phase model (post-MTZ to Week 4; left), continuation phase model (Week 4 to 8 to 12; middle), and follow-up phase model (Week 12 to 24; right), analogous to portrayal of LBP arm in **Figure 6A**.

medRxiv preprint doi: https://doi.org/10.1101/2025.08.18.25333897; this version posted August 21, 2025. The copyright holder for this preprint (which was not certified by peer review) is the author/funder, who has granted medRxiv a license to display the preprint in perpetuity.
It is made available under a CC-BY 4.0 International license .

Figure S9: Variables associated with microbiota categories in placebo recipients

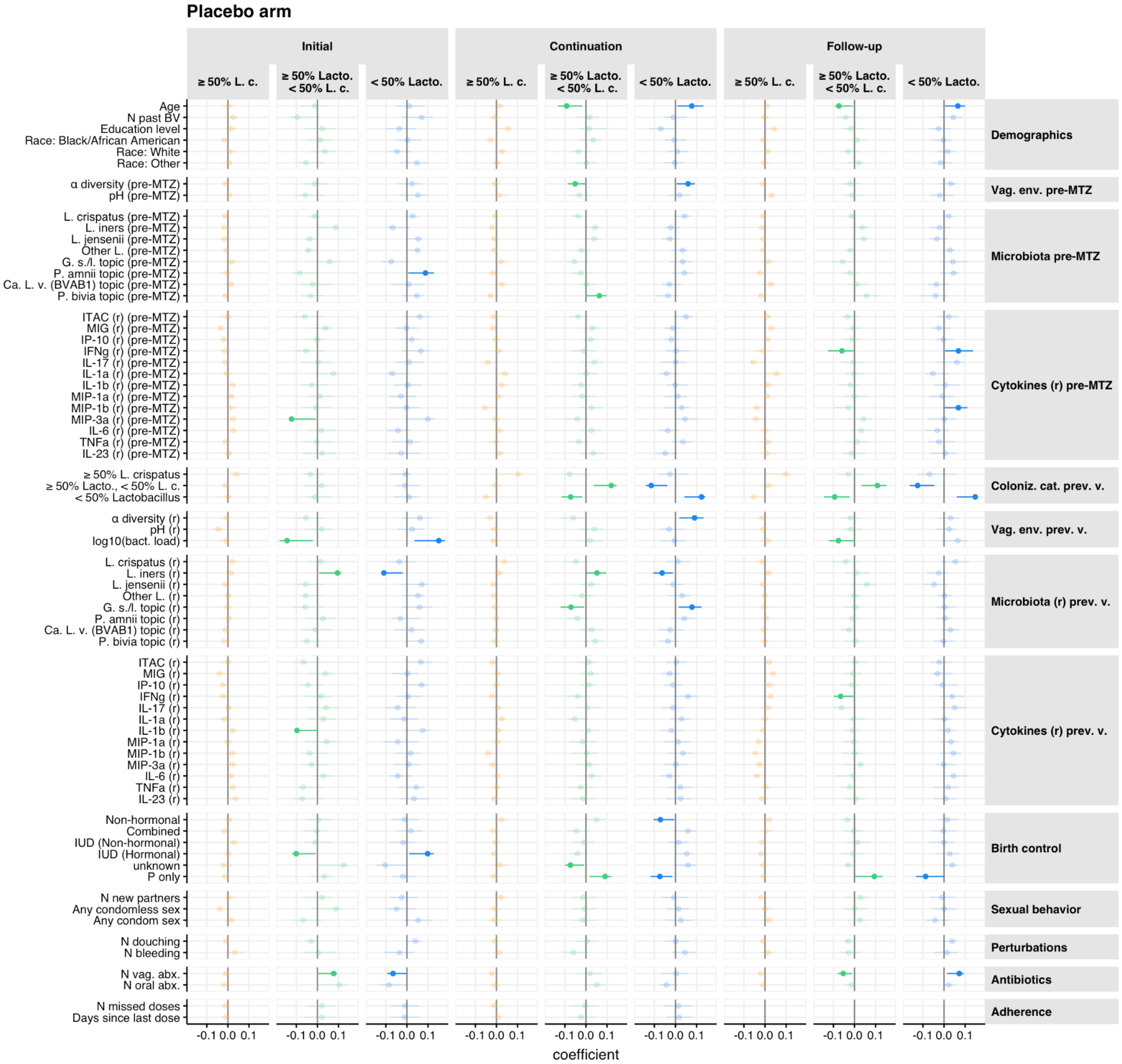

Coefficient of association (dots, x-axis) and associated bootstrapped 95% CI (horizontal segments) between explanatory variables (y-axis) and microbiota categories (colors, horizontal panels) for each phase of the trial (horizontal panels) when MB-PLS-DA models are fitted on the placebo arm participants' data. Variables whose 95% CI include 0 (suggesting no statistically significant associations) are shown

medRxiv preprint doi: https://doi.org/10.1101/2025.08.18.25333897; this version posted August 21, 2025. The copyright holder for this preprint (which was not certified by peer review) is the author/funder, who has granted medRxiv a license to display the preprint in perpetuity.
It is made available under a CC-BY 4.0 International license .

in transparent/lighter shades. Variables are shown grouped according to their assigned thematic blocks (**Figure 6A**, **S8C Table S6**).
